# Suicidality in the Criminal Justice System: The Role of Cumulative Adversity and Protective Factors

**DOI:** 10.3390/healthcare14020194

**Published:** 2026-01-13

**Authors:** Guilherme Welter Wendt, Kauê Furquim Depieri, Dalila Moter Benvegnú, Iara Teixeira, Patricia Silva, Felipe Alckmin-Carvalho

**Affiliations:** 1 Department of Medical Sciences, Postgraduate Program in Applied Health Sciences, Western Paraná State University, Francisco Beltrão 85601-970, Brazil; guilherme.wendt@unioeste.br (G.W.W.); kauedepierifurquim@gmail.com (K.F.D.); 2 Department of Biochemistry and Pharmacology, Federal University of Fronteira Sul (UFFS), Rodovia PR-182, Km 466, Realeza Campus, P.O. Box 253, Realeza 85770-000, Brazil; dalilabenvegnu@yahoo.com.br; 3 Department of Psychology, University of Minho, 4710-057 Braga, Portugal; 4 Department of Psychology and Education, Faculty of Social and Human Sciences, University of Beira Interior, Campus IV, 6200-209 Covilhã, Portugal; pg.silva@ubi.pt (P.S.); felipe.carvalho@ubi.pt (F.A.-C.)

**Keywords:** suicidal ideation, suicide attempt, incarcerated men, prison health, social determinants of health, mental health inequalities

## Abstract

**Background:** Incarcerated men experience disproportionately high levels of health inequities shaped by social determinants, including poverty, violence, family adversity, trauma, and limited access to healthcare. These long-standing disadvantages, added to the adverse conditions experienced in prisons, may be associated with elevated rates of suicidality in this population. This study examined the prevalence of suicidal ideation and lifetime suicide attempts among men deprived of liberty in Southern Brazil and investigated the role of cumulative adversities and current protective factors in these outcomes. **Methods:** A cross-sectional study was conducted with 496 incarcerated men. Participants completed a sociodemographic and background questionnaire assessing lifetime adversity (e.g., hunger, homelessness, sexual abuse, domestic violence, family substance dependence) and current protective factors in prison (e.g., family visits, education, leisure, physical activity, religion, positive self-perception). Cumulative adversity and protective factors were operationalized as composite indices. Logistic regression models tested whether cumulative adversities and protective factors were independently associated with suicidal ideation and suicide attempts. **Results:** Lifetime prevalence was 9.6% for suicidal ideation and 10.8% for suicide attempts. Cumulative adversities were associated with higher odds of both suicidal ideation (OR = 1.43; 95% CI = 1.11–1.84; *p* = 0.006) and suicide attempts (OR = 1.94; 95% CI = 1.50–2.52; *p* < 0.001). Protective factors were associated with lower likelihood of suicidal ideation (OR = 0.74; 95% CI = 0.58–0.96; *p* = 0.020) but were not significantly associated with suicide attempts. No significant interaction effects were observed, indicating that protective factors did not moderate the impact of adversity. **Conclusions:** Suicidal tendencies among incarcerated men were associated with cumulative structural and psychosocial adversities. Protective factors in prison were associated with lower odds of ideation but not attempts. These associations may inform person-centered and equity-oriented approaches and are consistent with the relevance of social determinants to mental health, although causal inferences are not supported by this project.

## 1. Introduction

Suicide is a serious global public health issue, both because of the magnitude of the number of deaths and because of the long-term psychosocial implications. At least 720,000 suicides occur worldwide each year, representing one of the largest preventable causes of death in the 21st century [1]. Although many countries have seen a reduction in suicide rates over the past few decades, regions such as the Americas have recorded a worrying increase, indicating changes in the epidemiological and social patterns associated with suicidal behavior [2]. These data reinforce the urgency of understanding the risk factors involved, especially in adult groups under extreme conditions of vulnerability.

Despite the fact that suicide can affect individuals of all ages, genders, and social classes, there are certain groups that are especially vulnerable due to social, economic, and clinical factors [3,4]. Among these, adult men stand out, who, in various international contexts, have suicide rates three or four times higher than those of women [5]. This pattern could be explained by several factors, such as greater frequency of use of lethal methods, low demand for mental health services, and sociocultural influences [6].

In addition, comorbidity between drug use and psychiatric disorders is very common and poses an additional challenge to prison systems, which are often unable to provide adequate treatment [7,8]. These conditions are strongly correlated with suicidal ideation: prisoners with a history of mental disorders are approximately three times more likely to have suicidal ideation throughout their lives, and approximately 2.7 times more likely to have suicidal ideation in prison [9]. Institutional factors such as overcrowding, neglect, solitary confinement, and limited meaningful social interactions accumulate psychological distress and increase the risk of suicide [10,11].

While clinical and institutional factors are important, there is an increasing amount of literature that suggests that suicidality among men in prison is also strongly influenced by social determinants of health [12,13]. Many people enter the prison system after experiencing prolonged structural disadvantage across the life course—food insecurity, homelessness, abuse in childhood or adulthood, exposure to domestic violence, or substance dependence in the family [9,14]. These aspects reflect structural, cumulative disadvantages that are indicative of ongoing forms of social marginalization that existed prior to incarceration, and they may contribute to the individual’s ongoing potential for psychological vulnerability. In contrast, some aspects of the prison environment may result in protective factors that include aspects such as partaking in work or educational programming, facilitating routine physical activity, opportunities for leisure, family visiting, and the overall ability to foster a positive sense of self [15]. Undoubtedly, these features may also afford the inmate a certain degree of structure, purpose, meaningfulness, and social connection, and thus lower the experience of distress. Nonetheless, despite their potential to be relevant, distal forms of adversity and proximal forms of protective experience are seldom considered in combination as predictors of suicidality among inmates.

Along with this, most of the research related to suicidality among incarcerated populations has continued to primarily focus on diagnosed mental disorders, or even acute psychiatric symptoms, rather than the life-course adversities that often precede imprisonment and substantially contribute to suicidal vulnerability.

On the other hand, some aspects of prison life—like access to work, education, physical activity, leisure, family visits, and opportunities to maintain a positive self-concept—may become protective factors [16]. However, evidence on these potential buffers remains limited and inconsistent, particularly in comparing suicidal ideation and lifetime suicide attempts.

In the Brazilian context, there is a notable scarcity of quantitative research examining both cumulative adversities and protective experiences as determinants of suicidality among incarcerated men. Given this context, this study sought to assess the prevalence of suicidal ideation and lifetime suicide attempts among men deprived of liberty in a prison in Southern Brazil, and to examine how cumulative lifetime adversities and current protective factors are associated with these outcomes. We hypothesized that higher levels of cumulative adversities would be associated with higher likelihood of both suicidal ideation and suicide attempts. Further, presence of protective factors (e.g., receiving visits from family members, taking part in leisure activities, among others) was expected to be associated with lower likelihood to suicidal ideation and attempts.

## 2. Materials and Methods

### 2.1. Method

This is an observational, cross-sectional study in which we assessed the prevalence of suicidal ideation and possible associated risk factors in a sample of incarcerated Brazilian men.

### 2.2. Setting

This study was conducted at the Francisco Beltrão State Penitentiary (DEPPEN-PFB) in 2023. It is a closed-regime penal establishment, intended for the custody of male prisoners who have already been convicted. Regarding healthcare and psychosocial support, the prison’s technical staff comprises social workers, a psychologist, a dentist, a nurse, and nursing technicians. Francisco Beltrão is a city in the southern Brazilian state of Paraná, with a population of about 102,312 inhabitants [17]. According to the 2023 Firjan Municipal Development Index [18] the municipality’s score was 0.8742 (high), ranking it among the 10 most developed municipalities in Brazil.

### 2.3. Ethical Procedures

This study is part of the research program of the Federal University of Southern Frontier (UFFS), Realeza campus. The research project was approved by the Research Ethics Committee of this educational institution, with approval number 3.439.697. All aspects of Resolution 466/2012 of the National Health Council of the Brazilian Ministry of Health [19] were followed, and the data collection procedures, including interviews and the filling out of psychological assessment instruments were authorized by the inmates through the signing of the informed consent form. Given the sensitive nature of some of the questions, the prison’s staff were notified about cases requiring assistance, including psychiatric care.

### 2.4. Participants

The final sample included 496 participants, 41.33% of the 1200 individuals deprived of liberty at this DEPPEN institution. The sample size was calculated using Open Source Epidemiologic Statistics for Public Health, which indicated that 352 participants were needed to achieve 99.9% power, considering an overall presence of suicide ideation of 9 ± 5% and an alpha < 0.05 [20].

### 2.5. Instruments

Sociodemographic and Background Questionnaire:

It included direct questions about sociodemographic characteristics (age, education level, and marital status), criminal history (recidivism). Participants also answered yes/no questions regarding a set of lifetime adversity experiences, protective factors, and Suicidal behavior:

Suicidal ideation and suicide attempts were assessed using two single-item binary indicators assessing lifetime prevalence.

Suicidal Ideation: Participants were asked, “Have you ever seriously thought about killing yourself?” (0 = No, 1 = Yes).

Suicide Attempt: Participants were asked, “Have you ever tried to kill yourself?” (0 = No, 1 = Yes). These items capture the occurrence of suicidal thoughts or acts at any point in the respondent’s life, encompassing events both prior to incarceration and during the current sentence.

Lifetime exposure to adverse experiences:

Food Deprivation: History of frequently going hungry during childhood due to lack of food.

Homelessness: History of living on the streets/homelessness at any point in life.

Sexual Abuse: Self-reported history of being a victim of sexual abuse (childhood or adulthood).

Witnessed Domestic Violence: History of witnessing physical violence perpetrated by the father (or stepfather) against the mother during childhood.

Family Substance Abuse: History of alcohol or drug dependence among immediate family members.

Current protective and contextual factors within the prison environment:

Family Visits: Receives regular visits from family members.

School/Education: Currently attends prison school or educational classes.

Leisure Activities: Participates in organized leisure or artistic activities.

Religious Practice: Currently practices a religion or attends religious services.

Physical Activity: Engages in regular physical exercise/sports.

Positive Self-Perception: Responded affirmatively to the item “Do you like yourself?” (Self-esteem proxy).

### 2.6. Procedures

To conduct the research, the project was presented to the management of DEPPEN in Francisco Beltrão, which authorized the collection of data on the premises of that institution. Subsequently, the project was submitted to and approved by the Ethics Committee of the Federal University of Fronteira Sul (UFFS), to which the researchers are affiliated. After approval by the Ethics Committee, contact was made with the management of the prison to arrange schedules and procedures to be adopted during the research. Thus, with the assistance of the prison management, an initial screening was carried out, according to the inclusion/exclusion criteria, for the selection of participants. The inclusion criteria comprised males aged 18 to 70 years who had been incarcerated at the Francisco Beltrão State Penitentiary for at least six months. Exclusion criteria included the inability to read or write and refusal to participate in the study. Notably, no potential participants were excluded based on levels of literacy criteria. All individuals recruited demonstrated sufficient reading skills to complete the instruments.

In a second stage, data collection began, in which the individuals evaluated were selected by prison officials so as not to alter the routine and movement of the location. A maximum of 21 inmates were moved to one of the prison classrooms to answer the questionnaire. As suggested by prison staff, we opted to limit the number of participants to 21 in order to ensure that the classroom provided enough personal space for comfort and data privacy.

### 2.7. Data Analysis

Data were analyzed using SPSS (version 31). Sociodemographic, contextual, and clinical characteristics were summarized using descriptive statistics (frequencies, percentages, means, and standard deviations). Missing data were handled using listwise deletion for the regression analyses; specifically, participants with incomplete data on the predictor indices or outcome variables were excluded, resulting in a final analytical sample of 440 (88.7% retention rate).

Before the main analyses, all lifetime adversity and protective/contextual items were recoded into binary variables (0 = no, 1 = yes). These variables were then combined into two composite indices.

Cumulative adversity index (0–5)

Included the presence of the following lifetime adversities:

- Food Deprivation
- History of homelessness
- History of Sexual Victimization
- Witnessed Domestic Violence
- Family history of alcohol or drug dependence

Note: Higher scores indicate greater cumulative adversity.

Protective factors index (0–6)

Included the presence of the following protective/contextual elements within the prison environment:

- Receiving family visits
- Attending the prison school
- Engaging in leisure activities
- Having a religion
- Engaging in physical activity
- Positive self-perception (“liking oneself”)

Note: Higher scores indicate a greater number of protective factors.

We measured cumulative adversity and protective factors using an unweighted index, drawing directly from the Cumulative Risk [21]. The argument is that the total count of risk exposures is more strongly associated with outcomes than the severity of any single factor. We favored this metric for its pragmatism. Unlike weighted schemes, which often require assumptions about relative severity that are difficult to justify, unweighted indices are robust to collinearity. They remain statistically sensitive even in smaller samples where power is a concern.

Due to the limited number of outcome events (n = 43 for ideation and n = 48 for attempts), we prioritized parsimonious models to maintain a stable Events Per Variable (EPV) ratio and avoid overfitting [22]. Preliminary bivariate checks indicated that sociodemographic covariates (age, education, marital status, recidivism) were not significantly associated with the outcomes (*p* > 0.30) and were therefore excluded from the final models. Additionally, we conducted bivariate analyses of individual index components and a sensitivity analysis excluding the high-prevalence item “liking oneself” to test the robustness of the findings.

The linearity assumption for the continuous cumulative indices was verified using the Box–Tidwell test (assessment of interaction terms between predictors and their natural logs). Model fit and performance were evaluated using −2 Log Likelihood, Nagelkerke R2, the Hosmer–Lemeshow test for calibration (*p* > 0.05 indicates adequate fit), and the Area Under the Receiver Operating Characteristic Curve (AUC) for discrimination. Multicollinearity diagnostics showed no problematic values (VIF < 2). Odds Ratios (OR), 95% confidence intervals (CI), and Wald statistics were reported. Statistical significance was set at *p* < 0.05 (two-tailed).

## 3. Results

The sample for this study consisted of 496 adult men deprived of their liberty (mean age 31, SD = 8.0). Most had low levels of education, were single, and reported having experienced multiple adversities during their lives. More than half of the participants were repeat offenders in Brazilian penal institutions. In the current context, a large part of the sample reported having protective factors, such as participating in leisure or educational activities in prison, receiving visits, and having a religion.

The prevalence of suicidal ideation was 9.6% and suicide attempts throughout life were around 10.8% for our sample. The details can be seen in Table 1.

To examine whether lifetime adversity and current protective factors were associated with suicidal ideation and/or attempts, we performed logistic regression analyses. First, with suicidal ideation as the dependent variable, the analysis showed that greater cumulative adversity was associated with higher odds of suicidal ideation, while protective factors were associated with lower odds of ideation. Each additional adversity was associated with 43% higher odds of suicidal ideation, while each additional protective factor was associated with 26% lower odds (Table 2). The model demonstrated a Nagelkerke R2 of 0.130, indicating that the predictors explain approximately 13% of the variance in suicidal ideation. The model showed adequate calibration (Hosmer–Lemeshow test *p* = 0.325) and fair discrimination (AUC = 0.67). Linearity of the continuous indices was confirmed via the Box–Tidwell test (non-significant non-linear terms).

Bivariate analyses of individual components (Supplementary Table S1) provided further context: among risk factors, sexual abuse had the strongest association (OR = 5.61), while among protective factors, ‘liking oneself’ had the strongest association. However, other items such as physical activity also showed associations with lower odds of ideation. A sensitivity analysis excluding the high-prevalence item ‘likes oneself’ confirmed the robustness of the cumulative protective index, which remained statistically significant (*p* < 0.05) even without this item.

Regarding suicide attempts (Table 3), only the presence of accumulated adversities was significant in its association with attempts, suggesting that each additional adversity practically doubles the odds of a suicide attempt (94%). The model demonstrated good discrimination (AUC = 0.72) and calibration (Hosmer–Lemeshow test *p* = 0.511), with linearity assumptions met. The explanatory power of this model was higher, with a Nagelkerke R2 of 0.189 (approx. 19% of variance explained), indicating a moderate fit given the multifactorial nature of suicidal behavior.

## 4. Discussion

In this study, we investigated the prevalence of suicidal ideation and suicide attempts in a sample of Brazilian men deprived of liberty in a penitentiary located in a medium-sized city in southern Brazil. In addition, we examined how accumulating adversities and current protective experiences were associated with suicidal ideation and suicide attempts among participants. Importantly, outcomes refer to lifetime suicidal ideation and lifetime suicide attempts (i.e., at any time in life), not necessarily occurring during incarceration.

The results revealed a clear graded pattern: the accumulation of lifetime adversities was associated with progressively higher odds of both suicidal ideation and previous suicide attempts, whereas protective factors were associated with lower odds of ideation but not with attempts. Taken together, these findings suggest that suicidality among incarcerated men co-occurs with long-term structural disadvantages, alongside the availability or absence of proximal psychosocial supports during imprisonment.

The prevalence of suicidal ideation and attempts among incarcerated men is a well-documented phenomenon in the literature. Suicidality among incarcerated men is considerably higher than in the general population. Studies such as that by Fazel point to a risk 3 to 10 times higher than the general population, depending on the country studied [9]. The meta-analysis by Favril et al. [12] analyzed the prevalence of suicide attempts in 20 studies with data from 20 countries. Based on a sample of 12,269 incarcerated men, a prevalence of 8.6% (95% CI: 6.1–11.2) was found, a value close to that observed in this study.

Although incarceration has been associated with elevated suicide risk in prison populations, after analysing data from 24 countries, the study by Fazel et al. [9] found no isolated association between prison system issues and suicide risk, assuming that among the participants analyzed, suicide risk was the result of a complex interaction of individual, environmental and psychosocial variables. The model proposes that suicide vulnerability among people deprived of liberty is partly related to social and health inequalities individuals are exposed since birth, which increases their risk of ultimately ending up in prison [23]. In addition, prior studies describe prisons as disproportionately housing highly marginalized individuals, who have life stories marked by chronic stressors such as poverty, parental neglect, family psychopathology, domestic violence, social isolation, and loneliness and trauma, as well as the use of maladaptive coping strategies such as alcohol and other drug abuse, which increase the risk of problems with the law [23,24]. Consistent with this model, we found high prevalence of adversity in our sample. In this context, it is possible that the social determinants that increase the likelihood of these men committing crimes may also increase their likelihood of suicide [25,26].

Brazil is characterized by profound social inequalities that are mirrored within its correctional system. The prison environment often amplifies these pre-existing structural vulnerabilities, such as poverty and lack of access to services, creating a continuum of disadvantage that exacerbates psychological distress [27,28]. In our study, the creation of the composite demonstrated a pattern of increased ideation and attempts with each added adversity. Studies corroborate this finding, showing that the greater the accumulation of adversities throughout life, the greater the vulnerability to mental disorders, self-destructive behaviors, and suicide [29,30]. and that these associations may persist across the life course, even if it was experienced during childhood [12,24].

This situation reflects the functioning of racism as a cross-cutting determinant of social and emotional adversities which [13], accumulated over the course of a lifetime, increase the risk of psychological distress and suicidality [31,32]. On the other hand, in our sample, the cumulative presence of protective factors during incarceration was associated with lower suicidal ideation but was not significant in reducing suicidal attempts. Considering the protective variables studied, receiving visits from family members is one of the most consistent in the literature [33,34]. Furthermore, we believe that structured activities in the prison context, such as attending school or participating in leisure activities, may reduce suicidal ideation by decreasing idleness and mental rumination. Together, these elements may have formed protective microsystems that, while not eliminating the risk, reduced the intensity of ideational processes related to self-harm, for example, by reinforcing feelings of belonging, autonomy, purpose, and personal value, even in a context of deprivation.

This finding aligns with People-Centered Approaches to correctional mental health, which emphasize individualized, collaborative, and dignity-based care focused on each person’s needs, strengths, and participation in rehabilitation, consistent with the World Health Organization framework for people-centred care [35] and other studies [36,37].

Crucially, the link between protective factors and suicidality appears stage-dependent. Our analysis showed a clear association with reduced ideation, but no such link existed for suicide attempts. This suggests that the ‘buffering’ capacity of social or institutional support may be limited to the psychological sphere of ideation. Among actual attempts, cumulative adversity is the more significant predictor. The implication is that for high-risk inmates, the presence of protective factors does not necessarily negate the strong association between accumulated adversity and self-harm behavior [38].

Linked to this is the Brazilian prison system itself, which is characterized by chronic overcrowding, structural neglect, and high levels of violence, which exacerbates the physical and mental suffering of inmates [39]. Overcrowding, combined with the control of facilities by criminal factions and recurring riots, reflects the collapse of state governance and the failure of prison management. These conditions not only violate basic human rights but also amplify psychological suffering and impede social reintegration.

### 4.1. Implications and Recommendations

From an ecological perspective, the findings suggest that suicide prevention among incarcerated men should be considered both within correctional settings and in relation to pre-incarceration vulnerabilities, with potential implications for the organization of care in the prison system, for public policies, and for professionals who work with this population.

Within correctional settings, the results support strengthening proximal protective resources included in our protective factor index, given their association with lower suicidal ideation. Improving the provision of psychological/psychiatric care and integrating screening and follow-up actions may be particularly relevant for individuals with higher cumulative adversity, considering the consistent association of this dimension with ideation and attempts [35].

Implementing these principles entails organizational and psychosocial changes to prison routines. From an organizational perspective, this may include a more humane environment and access to necessary health services, as well as training for correctional staff and teams in human rights, communication, and suicide prevention [40,41]. From a psychosocial perspective, prison programs can expand opportunities for participation in productive and meaningful activities (e.g., education, vocational training, and sports), which relate to a sense of agency and social connection [42,43]. Likewise, facilitating contact with family members and peers, as well as access to spiritual/religious programs, may foster bonds and hope, components compatible with the protective factors assessed [43,44,45]. Individualized care plans integrating mental health and rehabilitation can promote autonomy and engagement in the recovery process, aligning with the logic of people-centred care [42].

Beyond prison, the findings are consistent with the relevance of upstream social determinants and structural inequities as correlates of vulnerability, given that cumulative adversity was strongly associated with both outcomes [46,47]. Thus, equity-oriented policies aimed at reducing exposure to severe adversities (e.g., food insecurity, homelessness, and violence) may be relevant to the broader prevention landscape. Finally, strengthening continuity of care before, during, and after imprisonment through coordination between justice, health, and social assistance sectors is consistent with WHO frameworks and the United Nations Standard Minimum Rules for the Treatment of Prisoners [48,49].

### 4.2. Limitations and Future Directions

This study has limitations that should be acknowledged. First, the cross-sectional design precludes causal and temporal inference regarding the relationships between cumulative adversity, protective factors, and suicidality. Second, measures relied on retrospective self-report, which may introduce recall and reporting biases, particularly for lifetime suicidal ideation/attempts and early adversities.

Third, the study did not establish the timing of suicide attempts relative to incarceration (i.e., pre-incarceration vs. during custody), limiting interpretation of how institutional exposure relates to suicidal behavior. Fourth, some contextual variables were not assessed (e.g., race/ethnicity, sexual orientation, gender identity, solitary confinement, and other institutional exposures), which may contribute to residual confounding. Our outcome measures lack granularity. By opting for single-item binary measures for suicidality, we missed details regarding severity, frequency, and intent.

Participants were recruited from a single prison located in a relatively socioeconomically developed region, which may limit the generalizability of the findings to other prison contexts in Brazil. This is particularly relevant given that Brazil is the fifth largest country in the world in terms of land area and is extremely heterogeneous in terms of social and economic conditions. Moreover, the process of selecting participants was mediated by prison staff to preserve institutional security and routine policies. This sampling strategy may result in selection bias, particularly because such procedures could likely contain inmates with better behavioral records or lower security classifications. Moreover, literacy necessary for participating would have precluded the most educationally disadvantaged individuals, casting some doubts on the generalizability of the results to the overall prison population. Finally, even though anonymity was guaranteed and the classroom used was private, the setting of an institution and the presence of authority figures during the movement of participants might have resulted in social desirability bias.

Despite these limitations, the study provides novel, contextually grounded evidence from a population that remains underrepresented in international research. To deepen understanding of these findings, future investigations should employ longitudinal and mixed-methods designs and include correctional facilities in other regions of Brazil—particularly areas with substantially lower socioeconomic development, such as the North and Northeast—to capture a broader range of incarceration contexts and vulnerabilities. Further research should also incorporate institutional indicators (e.g., cell conditions, disciplinary measures, and access to health services) and examine intersectional vulnerabilities, including race, sexual orientation, and socioeconomic background. In addition, intervention and implementation studies are needed to determine whether person-centered, trauma-informed strategies and the strengthening of specific protective resources are associated with sustained reductions in suicidality over time.

## 5. Conclusions

This study shows that cumulative lifetime adversity (composite index) is associated with higher odds of both lifetime suicidal ideation and lifetime suicide attempts among incarcerated men, whereas in-prison protective resources (composite index) are associated with lower odds of ideation but not attempts. The findings highlight a graded pattern of association with adversity and a differential pattern for protective resources across suicidality outcomes. Together, these results contribute to evidence on suicidality in prison populations by identifying profiles of vulnerability and protection that may support people-centered approaches to assessment and prevention.

More broadly, the pattern observed is consistent with suicidality being intertwined with upstream social determinants and structural inequities that shape vulnerability before and during imprisonment. In this sense, prison mental health and suicide prevention can be situated within a wider public health perspective that connects correctional settings with community conditions and continuity of care.

## Figures and Tables

**Table 1:** Sociodemographic and Background Characteristics of the sample.

Variable	Category	n	Valid %
Age (categorical)	18–24	93	18.8
	25–34	219	44.2
	35–44	88	17.7
	45–54	23	4.6
	55+	73	14.7
	Total valid	496	100.0
Education level	Low education (basic or less)	277	62.2
	Middle school or higher	168	37.8
	Total valid	445	100.0
Marital status	Single	271	60.4
	Married/cohabiting	129	28.7
	Divorced/separated	46	10.2
	Widowed	3	0.7
	Total valid	449	100.0
Reincident (n = 434)	Yes	248	57.1
	No	186	42.9
Food Deprivation	Yes	190	43.0
	No	252	57.0
	Total valid	442	100.0
Homelessness history	Yes	99	22.6
	No	340	77.4
	Total valid	439	100.0
History of Sexual Victimization	Yes	32	7.3
	No	404	92.7
	Total valid	436	100.0
Witnessed Domestic Violence	Yes	179	40.6
	No	262	59.4
	Total valid	441	100.0
Family substance dependence	Yes	283	64.8
	No	154	35.2
	Total valid	437	100.0
Receives family visits	Yes	217	49.3
	No	223	50.7
	Total valid	440	100.0
Attends prison school	Yes	165	37.8
	No	272	62.2
	Total valid	437	100.0
Engages in leisure activities	Yes	226	52.9
	No	201	47.1
	Total valid	427	100.0
Has a religion	Yes	317	74.6
	No	108	25.4
	Total valid	425	100.0
Perceives self positively (“likes oneself”)	Yes	412	94.1
	No	26	5.9
	Total valid	438	100.0
Physical activity	Yes	253	60.2
	No	167	39.8
	Total valid	420	100.0
Suicidal ideation (lifetime)	Yes	43	9.6
	No	403	90.4
	Total valid	446	100.0
Suicide attempt (lifetime)	Yes	48	10.8
	No	398	89.2
	Total valid	446	100.0
Cumulative adversity index (0–5)	0 adversities	74	16.7
	1 adversity	132	29.7
	2 adversities	114	25.7
	≥3 adversities	124	27.9
	Total valid	444	100.0
Protective factors index (0–6)	0–1 protective factors	29	6.5
	2–3 protective factors	175	39.3
	≥4 protective factors	241	54.2
	Total valid	445	100.0

**Table 2:** Logistic regression examining associations with lifetime suicidal ideation from cumulative adversities and protective factors (N = 440).

Variable	B	SE	Wald	df	*p*	OR (Exp(B))	95% CI for OR
Cumulative Adversity Index (0–5)	0.36	0.13	7.48	1	0.006	1.43	[1.11, 1.84]
Protective Factors Index (0–6)	−0.30	0.13	5.38	1	0.020	0.74	[0.58, 0.96]
Constant	−2.00	0.55	13.43	1	<0.001	0.14	—

Note. Higher scores on the adversity index indicate more lifetime adversities. Higher scores on the protective factors index indicate more protective elements. B = regression coefficient, SE = standard error, Wald = Wald chi-square test, df = degrees of freedom, *p* = significance level, OR = odds ratio, CI = confidence interval.

**Table 3:** Logistic regression examining associations with lifetime suicide attempts from cumulative adversities and protective factors (N = 440).

Variable	B	SE	Wald	df	*p*	OR (Exp(B))	95% CI for OR
Cumulative Adversity Index (0–5)	0.66	0.13	25.15	1	<0.001	1.94	[1.50, 2.52]
Protective Factors Index (0–6)	−0.14	0.12	1.39	1	0.239	0.87	[0.68, 1.10]
Constant	−3.11	0.58	28.92	1	<0.001	0.05	—

Note. Odds ratios (ORs) represent the likelihood of reporting a past suicide attempt for each 1-unit change in the predictor. B = regression coefficient, SE = standard error, Wald = Wald chi-square test, df = degrees of freedom, *p* = significance level, OR = odds ratio, CI = confidence interval.

## Data Availability

Data can be obtained from the authors upon reasonable request due to ethical restrictions.

## Supplementary Materials

The following supporting information can be downloaded at: https://www.mdpi.com/article/10.3390/healthcare14020194/s1, Supplementary Table S1: Bivariate associations between individual adversity/protective factors and suicidality.
