# Supplementary material for: Suicidality in the Criminal Justice System: The Role of Cumulative Adversity and Protective Factors
Source: Healthcare (Basel). 2026 Jan 13;14(2):194. doi: 10.3390/healthcare14020194 (PMC12840824; doi:10.3390/healthcare14020194)
Supplement: Supplementary file 1 [file healthcare-14-00194-s001.zip › healthcare-4046902-supplementary.pdf]

**Supplementary Table S1 Bivariate associations between individual adversity/protective factors and suicidality.**

| Factor | Suicidal IdeationOR [95% CI] | p-value | Suicide AttemptOR [95% CI] | p-value |
|---|---|---|---|---|
| **Adversity Factors** | | | | |
| Childhood Hunger | 1.63 [0.85, 3.10] | .185 | **3.09 [1.62, 5.92]** | **.001** |
| Homelessness | **3.22 [1.65, 6.29]** | **.001** | **3.50 [1.85, 6.61]** | **< .001** |
| History of Sexual Victimization | **5.61 [2.43, 12.92]** | **< .001** | **3.85 [1.66, 8.93]** | **.002** |
| Witnessed Dom. Violence | 1.03 [0.54, 1.99] | >.99 | **2.26 [1.21, 4.20]** | **.014** |
| Family Substance Abuse | 1.37 [0.68, 2.77] | .475 | **2.85 [1.29, 6.27]** | **.011** |
| **Protective Factors** | | | | |
| Family Visits | 0.88 [0.46, 1.67] | .814 | 0.62 [0.33, 1.16] | .181 |
| School/Classes | 0.52 [0.25, 1.09] | .112 | **0.42 [0.20, 0.87]** | **.026** |
| Leisure Activities | 0.69 [0.35, 1.35] | .364 | 0.92 [0.50, 1.71] | .917 |
| Religious Practice | 1.40 [0.63, 3.15] | .525 | 0.85 [0.43, 1.68] | .772 |
| Physical Activity | **0.37 [0.18, 0.74]** | **.006** | 1.10 [0.58, 2.08] | .894 |
| Likes Oneself | **0.23 [0.09, 0.60]** | **.004** | **0.21 [0.09, 0.52]** | **.001** |

**Note.** OR = Odds Ratio; CI = Confidence Interval. Values in bold indicate statistical significance ($p < .05$). Unadjusted logistic regression models.
